# A Randomised Controlled Phase II Trial of the Combination of XELOX with Thalidomide for the First-line Treatment of Metastatic Colorectal Cancer

**DOI:** 10.3969/j.issn.2095-3941.2012.02.005

**Published:** 2012-06

**Authors:** Jing Lv, Ning Liu, Ke-wei Liu, Ai-ping Ding, Hao Wang, Wen-sheng Qiu

**Affiliations:** Department of Oncology, The Affiliated Hospital of Qingdao University Medical School, Qingdao 266003, China

**Keywords:** colorectal neoplasm, thalidomide, oxaliplatin, capecitabine

## Abstract

**Objective:**

To evaluate the efficacy and safety of the combination of XELOX regimen (oxaliplatin plus capecitabine) with thalidomide for the first-line treatment of metastatic colorectal cancer (MCRC).

**Methods:**

All of the 89 patients with MCRC who fulfilled eligibility criteria were randomly assigned to treatment group (*n*=44) and control group (*n*=45). The treatment group received a combination of XELOX with thalidomide and the control group received XELOX alone. Each patient received at least 2 cycles of treatment (1 cycle=21 d). The primary endpoint was progression-free survival (PFS) and the secondary endpoints were objective response rate (ORR) as well as disease control rate (DCR). Drug safety and quality of life were also assessed.

**Results:**

The median PFS of the treatment and control groups were 5.6 and 5.2 months, respectively. The difference did not have a statistical significance (*P*=0.307). The ORRs of the two groups also had no statistical difference (34.1% *vs.* 26.7%, *P*=0.446). The addition of thalidomide to XELOX significantly improved the DCR (63.6% *vs.* 42.2%, *P*=0.043). Among 24 patients with hepatic metastasis in the treatment group, 2 patients satisfied the surgical criteria after treatment but none of 23 patients in the control group did. Grade 3 or 4 constipation in patients treated with thalidomide was significantly increased (20.5% *vs.* 4.4%, *P*=0.022) but didn’t result in treatment interruption. The rate of lethargy was increased but the difference between the two groups had no statistical significance (13.6% *vs*. 4.4%, *P*=0.130). The quality of life had no statistical difference between the two groups.

**Conclusions:**

The combination of XELOX with thalidomide for the first-line treatment of MCRC was well tolerated. Statistically significant improvement was achieved for the DCR but not for PFS.

## Introduction

Chemotherapy combined with targeted drugs is the current standard management for disseminated metastatic colorectal cancer (MCRC). The baseline chemotherapeutic agents include oxaliplatin, irinotecan and fluorouracil. The combination of oxaliplatin with capecitabine, known as XELOX regimen, has been more easily accepted by patients because of its confirmed efficacy and convenience of use ^[^^1^^]^.

Targeted drugs such as tumor angiogenesis inhibitors and epidermal growth factor receptor inhibitors are expensive which limit their use. Thalidomide, a sedative and anti-inflammatory drug used in immunotherapy has become a research focus since the discovery of its anti-angiogenesis properties in recent years ^[^^2^^, ^^3^^]^. A randomized controlled phase II trial was undertaken in the oncology department of The Affiliated Hospital of Qingdao University Medical School to evaluate the efficacy and safety of the combination of XELOX with thalidomide for the first-line treatment of MCRC.

## Materials and Methods

### Eligibility criteria

Patients with pathologically confirmed MCRC were eligible for inclusion if they had at least one measurable target lesion; age 18-70 years; no previous treatment; Eastern Cooperative Oncology Group (ECOG) performance status 0-2; and a life expectancy greater than 3 months.

### General information

All of the 89 patients were enrolled since January 2007 to October 2009, and all of them provided informed consent. They were assigned to the treatment group (*n*=44) and the control group (*n*=45) by blocked randomization method. The baseline clinical characteristics of the two groups were comparable as summarised in **Table 1**.

**Table 1:** Baseline clinical characteristics of the two groups.

Item	Treatment group (*n*=44)	Control group (*n*=45)	*P*
ECOG PS score			
0	6	7	0.869
1	21	23	
2	17	15	
Primary tumor site			
Colon	18	21	0.584
Rectum	26	24	
Hepatic metastases			
Yes	24	23	0.746
No	20	22	
Pathology type			
Highly to moderately differentiated adenocarcinoma	12	14	0.691
Poorly differentiated or mucinous adenocarcinoma	32	31	
Adjuvant chemotherapy			
Yes	17	20	0.578
No	27	25	

### Therapeutic methods

The treatment group received XELOX combined with thalidomide, and the control group received XELOX alone. XELOX was given every 21 days for at least 2 cycles. Oxaliplatin, the trade name Aiheng (Oxaliplatin for Injection) from Jiangsu Hengrui Medicine Co., Ltd., 130 mg/m^2^ was given intravenously (iv.) for at least 2 h on day 1; Capecitabine, the trade name Xeloda from Shanghai Roche Pharmaceuticals Limited, 1 000 mg/m^2^ was given orally, twice daily on days 1-14. Thalidomide, obtained form Changzhou Pharmaceutical Factory, 200 mg was given orally, daily at bedtime on days 1-14, concurrently with chemotherapy and repeated every 21 days too. Tumor response was evaluated after each second chemotherapy cycle by thorough examination. The patients who had effective response and tolerable toxcity continued chemotherapy until disease progression or serious adverse reaction.

### Observation index

#### Clinical efficacy

There were 4 types of short-term efficacy according to the Response Evaluation Criteria in Solid Tumors version 1.1 (2009): complete response (CR), disappearance of all target lesions; partial response (PR), at least a 30% decrease in the sum of diameters of the target lesion, taking as reference the baseline sum diameters; stable disease (SD), the sum of the diameters of target lesions decreased no more than 30% or increased no more than 20%; and progressive disease (PD), at least a 20% increase in the sum of diameters of target lesions and an absolute increase of at least 5 mm or the appearance of new lesions. The definitions of the objective response rate (ORR) and disease control rate (DCR) are as follows: ORR (%) =(CR + PR) / total number of cases × 100%, and DCR (%) = (CR + PR + SD) / total number of cases × 100%.

The long-term efficacy for this trial was progression-free survival (PFS), defined as the time from the first administration of treatment medicine until confirmed PD or death from any cause.

#### Adverse reactions

Regarding drug safety, adverse reactions were evaluated according to the National Cancer Institute Common Terminology Criteria for Adverse Events (version 4.0).

#### Quality of life

The quality of life was assessed by the ECOG performance status scores.

### Statistical analysis

The comparisons of the baseline clinical characteristics, efficacy between groups, quality of life, and adverse reactions were performed using the Chi-square test. For the survival analysis, the log-rank method was adopted to test any difference. All data were processed by SPSS 11.5. *P*<0.05 was considered statistically significant.

## Results

### Treatment durations and clinical efficacy

The median durations of treatment of the two groups had no significant difference. The median duration of treatment of all patients was 6.2 (0.5–14) months and the median number of treatment cycles was 7.5 (1–17). The median follow-up time was 20.2 months. The short-term efficacy is shown in **Table 2**. The ORRs of the treatment and control groups were 34.1% and 26.7%, respectively, without statistical significant difference (*P*=0.446). The DCRs of the treatment and control groups were 63.6% and 42.2%, respectively, with a significant difference (*P*=0.043). In 24 cases with unresectable hepatic metastases in the treatment group, 2 patients had their hepatic metastases resected after the treatment and the resection rate was 8.3%. However, none of the 23 cases with unresectable hepatic metastases in the control group satisfied the criteria of resection after treatment. PFS of the treatment and control groups were 5.6 and 5.2 months, respectively, with no significant difference (*P*=0.307).

**Table 2:** Short-term efficacy of the two groups.

Group	Cases	CR	PR	SD	ORR	DCR
Treatment	44	1	14	13	15 (34.1)	28 (63.6)
Control	45	0	12	7	12 (26.7)	19 (42.2)

Note: Figures in parentheses were the percentage equivalent.

### Adverse reactions

Each case had at least one adverse reaction, and the most common adverse reactions included peripheral neurotoxicity, anorexia, fatigue, and hand-foot syndrome. Nevertheless, most of them were slight and well tolerated. **Table 3** summarised the grade III and IV adverse reactions. Among them, peripheral neurotoxicity and neutropenia were the most common toxicities in both groups, and the differences having a statistical significance. The rate of constipation of the treatment group was significantly higher than that of the control group (20.5% *vs*. 4.4%, *P*=0.022). The rate of drowsiness of the treatment group was also higher than that of the control group, but the difference was not significant (13.6% *vs*. 2.2%, *P*=0.130). There was not statistical difference between the groups in the other adverse reactions.

**Table 3:** Incidence rates of adverse reactions (Grade III and IV) of the 2 groups.

Adverse reactions	Treatment group (*n*=44)	Control group (*n*=45)	*P*
Peripheral neurotoxicity	8 (18.2)	10 (22.2)	0.635
Anorexia	2 (4.5)	5 (11.1)	0.250
Fatigue	4 (9.1)	6 (13.3)	0.526
Hand-foot syndrome	5 (11.4)	4 (8.9)	0.699
Neutropenia	7 (15.9)	9 (20.0)	0.615
Thrombocytopenia	3 (6.8)	5 (11.1)	0.479
Constipation	9 (20.5)	2 (4.4)	0.022
Drowsiness	6 (13.6)	2 (4.4)	0.130

Note: Figures in parentheses were the percentage equivalent.

### Quality of life

Patients’ quality of life based on the ECOG scores had no statistically significant difference between the two groups after treatment.

## Discussion

Colorectal cancer is the third most common cause of cancer-related death worldwide. The 5-year survival was only 8.1% in patients with MCRC ^[^^4^^]^. Evidence-based medicine has confirmed that chemotherapy results in improved overall survival of MCRC patients ^[^^5^^]^. XELOX is one of the accepted standard chemotherapy regimens at present ^[^^1^^]^. Different from FOLFOX (oxaliplatin plus calcium folinate/fluorouracil), XELOX used orally capecitabine instead of continous intravenous infusion fluorouracil. A randomized, double-blind, placebo controlled, phase III clinical trial (NO16966A) showed that the efficacy of XELOX as a first-line treatment for advanced colorectal cancer was non-inferior to FOLFOX4^[^^6^^]^. Another study showed that the medication convenience of XELOX was superior to that of FOLFOX6 ^[^^7^^]^, and its performance-cost ratio was also superior to FOLFOX4^[^^8^^]^. In this study, ORR of the patients receiving XELOX alone was 26.7% and PFS was 5.2 months, which were similar with previous findings. The most common adverse reactions of XELOX included peripheral neurotoxicity and hand-foot syndrome; the others were fairly slight. The majority of patients completed the established treatment plan in the outpatient department with good compliance.

A phase III randomized controlled trial showed that adding bevacizumab, an anti-angiogenesis drug, to the XELOX or FOLFOX chemotherapy improved PFS ^[^^9^^]^. The subgroup analysis of the study showed that improved PFS observed only in the group treated with bevacizumab plus XELOX, but not plus FOLFOX. This finding suggested that the efficacy of an anti-tumor angiogenesis agent combined with XELOX was superior to that with FOLFOX. However, bevacizumab was too expensive for many patients and it increased the risk of cerebral apoplexy and other arterial vascular events in senile patients. Most of colorectal cancer patients in China are elder. Therefore, bevacizumab was not widely used in the patients with MCRC in China.

Thalidomide was a glutamic acid derivative. Some *in vivo* and *in vitro* trials ^[^^10^^]^ have shown that thalidomide and its derivatives (lenalidomide and pomalidomide) have immuno-regulating, anti-angiogenesis, as well as anti-apoptotic efficacy. They could significantly inhibit the metastatic potential of a mouse colorectal cancer cell line. The addition of thalidomide to chemotherapy could improve the efficacy of hematological tumors and prolong PFS as well as overall survival ^[^^11^^]^. In May 2006, the Chinese Food and Drug Administration approved the application of thalidomide for treating multiple myeloma. The domestic and foreign studies on thalidomide for the treatment of solid tumors, particularly gastrointestinal tumors, were also growing ^[^^12^^-^^15^^]^. However, the conclusions from these studies were not consistent. Govindarajan ^[^^13^^]^ found that the combination of thalidomide with some other chemotherapeutic agents improved response rates in patients with metastatic and chemotherapy resistant colon cancer. However, a phase II clinical trial by McCollum et al. ^[^^14^^]^ showed Xeloda combined with thalidomide did not improve ORR of patients with progressive colorectal carcinoma. A domestic phase II clinical trial ^[^^15^^]^ showed that thalidomide combined with chemotherapy did not improve the ORR or PFS compared with chemotherapy alone but did decrease gastrointestinal reaction related to irinotecan, such as diarrhea, naupathia, etc.

This study investigated efficacy of XELOX combined with or without thalidomide for the first-line treatment of MCRC patients. ORR of the treatment group was significantly improved compared with the control group. Addition of thalidomide to XELOX could effectively control the disease, postponing time-to-progression. PFS of the treatment group (5.6 months) was higher than the control group (5.2 months), but without statistical difference. It is worth noting that among the patients with hepatic metastases, 2 patients in the treatment group got hepatectomy, and the hepatectomy rate was 8.3%; but none in the control group satisfied the criteria of hepatectomy. That suggested that further studies about combination of XELOX with thalidomide for the treatment of colorectal cancer patients with hepatic metastases are needed.

The rate of constipation was higher in thalidomide group compared with the control group, but that could be alleviated by adjusting the diet of the patient or orally administrating laxatives, and that didn’t lead to chemotherapy interruption. Thalidomide also caused drowsiness therefore patients must be instructed to take it at bedtime and not drive during the medication.

Thus, XELOX combined with thalidomide was well tolerated for the first-line treatment of MCRC. It can significantly increase DCR, although it fails to prolong PFS. A larger-sample clinical trial should be conducted in patients with hepatic metastases to explore whether the regimen could inprove the hepatectomy rate and overall survival.
